# Environmental and Economic Impact of an Innovative Biocide-Free Antifouling Coating for Naval Applications

**DOI:** 10.3390/ma16020748

**Published:** 2023-01-12

**Authors:** Simone Venettacci, Gennaro Salvatore Ponticelli, Flaviana Tagliaferri, Stefano Guarino

**Affiliations:** 1 Department of Engineering, University of Rome Niccolò Cusano, Via don Carlo Gnocchi 3, 00166 Rome, Italy; 2 Faculty of Engineering Sciences, Hochschule Mittweida University of Applied Sciences, Technikumplatz 17, 09644 Mittweida, Germany

**Keywords:** antifouling coating, life cycle costing, life cycle assessment, fuel saving, CO_2_ emission reduction, marine aquatic ecotoxicity, CML 2001 baseline method

## Abstract

The work provides an economic sustainability and environmental impact analysis for the validation of a biocide-free antifouling coating for marine applications able to reduce fuel consumption during navigation, CO_2_ emissions, and the overall environmental impacts associated with shipping, thanks to the reduction of incrustation and the avoidance of biocides release into the water. The results, related to the life cycle of the coating of a motor yacht, with an average sailing life of 25 years, show around 8.8% reduction in overall costs compared to a conventional paint, thanks to a more efficient antifouling action, which reduces the annual fuel consumption by ~13,700 kg/y, or ~9.6%. This leads to a reduction in CO_2_ emissions, associated with fuel consumption, of ~43.3 ton/y, as well as a lowering of the overall environmental impacts associated with the life cycle of the paint, by almost 10% for the most impactful damage classes, ensuring a greater environmental sustainability of the innovative coating, for the overall service life of the yacht on which it is applied.

## 1. Introduction

Antifouling coatings are commonly applied to the hull and propeller of boats to minimize or eliminate the deposition of marine organisms and algae. In fact, these naturally settle on the submerged surfaces of boats creating incrustation, or biofouling, that can damage the surface itself. Moreover, during navigation, the accumulation of biofouling slows down the boat with a consequent increase in fuel consumption, therefore considered an urgent environmental and economic concern [1,2].

From an economic point of view, the application of the antifouling coating entails several expenses, such as the cost of maintenance of the boat, the cost related to the cleaning of the hull, the costs of removing the paint, and the cost of the paint itself. At the same time, the application of the coating is necessary to avoid decreasing the speed of the boat, for a personal safety factor and to consume less fuel. From an environmental point of view, however, the use of antifouling products represents a problem for the marine environment as they contain biocides, i.e., toxic substances that prevent the formation of incrustations, but at the same time pollute the marine environment [2]. In fact, these biocidal toxins can slow down the proliferation of parasites such as algae, silt, coralline and other species, thus preventing the formation of biofouling on the hull of boats. These additives, in addition to being highly toxic, are also poorly biodegradable, therefore they can remain in marine sediments for a long time [3]. In this context, more eco-friendly alternatives are required.

New antifouling biocides are transforming into less toxic products [4], with a low tendency to bioaccumulate [5], and a low toxicity to non-target organisms [6,7]. However, most of the research works focused on the evaluation of the chemical, physical, and mechanical properties of antifouling coatings without considering their environmental and economic impact [8]. Among these, most of the studies investigated the effect on fisheries [9,10] and maritime transport [11,12], while there are not comprehensive studies that evaluate the environmental and economic impacts of antifouling paints only.

The choice of the right antifouling coating depends on several factors, e.g., type of boat, type of water, use, and frequency. It is therefore necessary to consider whether it is a fast boat rather than a slow one, if it is used frequently or occasionally, if it remains in the water all year round, or if it is stored appropriately during the winter. In general, antifouling paints can be divided into two distinct groups based on their composition: (i) self-polishing and (ii) hard-matrix paints. The self-polishing antifouling paints, also defined as water-soluble, self-cleaning, or ablative, have a combined chemical/mechanical action which increases their effectiveness [13]. The chemical action of the water and the mechanical action of the movement of the boat regenerate each coat of antifouling applied [14]. This type of paint has been designed to wear out during use, while keeping a new and smooth surface, against any type of marine incrustation. In fact, the water is gradually absorbed causing a gradual dissolution of the matrix. It is important to specify that the renewal takes place in the order of microns so there is no risk that the paint is completely consumed during the season. This feature makes self-polishing antifouling paints unsuitable for extremely fast boats. Furthermore, due to their progressive thinning during navigation, the removal of the remaining layers at the end of the season will be much easier. The advantages of applying this type of paint are the excellent protection of the submerged surface thanks to the combined chemical and mechanical action and easy removal using a pressure washer [14]. On the other hand, hard-matrix antifouling paints act only chemically. Being based on a polymeric binder, they are very resistant to abrasion and for this reason they represent an excellent solution for fast boats or frequently hauled or wheeled ones [15]. Moreover, they generally have lower costs than self-polishing antifouling paints and, as they suffer less from changes in water conditions, they are also suitable for long navigations [16].

Among the strategies to produce an effective and eco-sustainable antifouling marine paint, amphiphilic coatings represent the most promising solution [17,18]. However, most of the compositions contain fluorinate species for the formation of the hydrophobic moieties [19,20], which are potentially toxic for the marine environment [21,22]. Moreover, the procedures adopted to produce such coatings are very complicated and time-consuming, therefore preventing their implementation in a proper paint product [23]. To simplify the procedure, different alternatives have been proposed so far, e.g., by introducing surfactants into coating systems [24], by using polyurethanes [25], or through sol-gel processes [26]. While the first two procedures still require long time and high temperature, sol-gel chemistry can provide a fast and easy to implement alternative [27].

In this context, the present study aims at proposing the evaluation of the environmental and economic impact of an innovative hard-matrix amphiphilic formulation prepared via a sol-gel process [23]. The authors evaluated different strategies to manufacture amphiphilic coating systems, verifying the feasibility of the proposed method for the manufacturing of anti-fouling paints, through wettability analysis, as well as through an assessment of anti-fouling activity of the coatings [18,23]. In particular, previous works verified the effective antifouling/foul-release action of the coatings using a natural protein probe, namely white egg [18,23], as well as testing them against marine organisms, such as mussels and algae [17]. The excellent results obtained in tests against amphiphilic proteins, such as white egg, well simulated the behavior of amphiphilic gluing proteins used by most of marine organisms, animal or vegetal, to adhere to the surfaces of a hull, thus verifying the effectiveness of the innovative amphiphilic coatings developed [17,23].

The next step, here investigated, concerns the life cycle assessment and the life cycle costing evaluation of the proposed coating intended for naval applications. To this end, the first activity involved the study of the materials, energy, and waste flows, associated with each phase of the life cycle, i.e., paint production, application, navigation, and disposal. Then, an environmental assessment was performed in order to verify the eco-compatibility of the innovative solution, evaluating the savings in terms of fuel consumption, linked to the increased efficiency of the antifouling action, which improves boat’s hydrodynamic performance, and comparing both its life cycle and CO_2_ production with the traditional solution. Finally, an economic assessment of the production, application, navigation and disposal costs, for a diesel-powered motor yacht, was completed, in order to evaluate the economics benefits linked to fuel consumption reduction and biocides absence during all the life cycle.

## 2. Materials and Methods

The present work aims at comparing, from both the economic and the environmental point of view, the impact of an innovative biocide-free antifouling coating for naval applications, named EXP in the following, against the traditional commercial paint “Unigloss” sold by Veneziani Yachting (Boero, Genoa, Italy) [28], with the addition of two biocides, i.e., tralopyril [29] and zinc pyrithione [30], named TRD. The following sections highlight the main characteristics. However, it is worth mentioning here that the innovative biocide-free coating has been previously demonstrated to have antifouling properties against amphiphilic proteins, using white egg as testing probe [18,23], as well as testing them against marine organisms [17], thus proving the effectiveness of the innovative hard-matrix amphiphilic formulation in producing a robust antifouling coating [23].

The Life Cycle Assessment (LCA) and the Life Cycle Costing (LCC) methodologies have been considered to evaluate the environmental and economic negative impacts and benefits in decision-making processes towards a more sustainable antifouling paint throughout its life cycle.

The LCA is a methodology that allows to record, quantify, and evaluate the environmental footprint of a specific product or service along its entire life cycle. It is a standardized procedure [31,32] that examines the entire life of a product “from cradle to grave” [33]. This therefore means that the entire process is analyzed starting from the extraction of raw materials up to their disposal, through their use and consumption [34]. The same procedure is applied through the LCC methodology [35], but with the aim of evaluating the costs associated with the life cycle of the product.

### 2.1. Life Cycle Assessment

The LCA analysis has been carried out by using the software SimaPro^®^ 7.1. The latter allows the creation of models related to the entire life cycle of a product with the aim of analyzing and quantifying the environmental impact. Moreover, it is possible to compare the results with those of other products, identifying any critical issue within the life cycle, through CML 2001 baseline method.

In general, the Life Cycle Assessment methodology is divided into four phases, which will be analyzed in detail in the following sections:

1. definition of scope and objective,
2. inventory analysis,
3. assessment of potential environmental impact,
4. interpretation and improvement of results.

#### 2.1.1. Definition of Scope and Objective

In this first phase, the most important decisions are made and the study of the entire LCA analysis is planned. To implement the analysis, three main items must be defined:

5. the target of the study, which in this case is to analyze the environmental sustainability of two antifouling paints, whose function is to protect the hull of boats from any biological incrustations;
6. the functional unit, which is chosen as the painting coverage of 1 m^2^ of hull;
7. the system limits, which correspond to the entire hull coating life cycle, therefore starting from the extraction of raw materials up to the disposal of waste.

It is important to underline that for the operational phase of the paint, 500 h of use of the boat per year will be considered and that the application of the two coatings was performed on the same boat, i.e., a motor yacht, 23.6 m long, 5.5 m wide, with a hull surface of 105 m^2^.

#### 2.1.2. Inventory Analysis

In this second phase, the flows of materials and energy (input) and waste (output) are defined, taking into consideration the entire life of the products and building an appropriate model. Specifically, the following will be analyzed:

- the stage of production of 1 kg of paint;
- the painting process of 1 m^2^ of hull;
- the annual fuel consumption for a total of 500 h of navigation.

##### Production Process

Table 1 and Table 2 report the formulations used to produce 1 kg of paints, respectively for the innovative and the traditional coatings. Each material of the two formulations corresponds to a specific item within the SimaPro^®^ 7.1 software database.

##### Painting Process

For the application of the coatings, data are calculated considering a surface of 1 m^2^ of hull, and that the innovative painting is applied twice per year while the traditional one only once per year. Seven steps were involved to create the process trees, which were applied with the same procedures and on the same vessel:

8. the boat is transported on land with consequent fuel consumption, and the hull is cleaned using a pressure washer, with consumption of water and low voltage (LV) electricity;
9. the old paint is removed with an orbital pneumatic sander, which involves the use of both the compressed air machine and the use of abrasive paper discs;
10. the surface is prepared for the application of the new antifouling coating. The same sander as the previous step is used, which involves the use of both the compressed air machine and the abrasive paper discs;
11. the parts of the boat that do not require the coating are covered with paper tape and plastic sheets;
12. the primer and a thinner are applied to the hull of the boat using a spray gun;
13. the hull of the boat is coated with antifouling paint by using the same spray gun of the previous step;
14. the boat is brought back to the sea, ready for sailing.

Table 3 reports the items consumed during the seven steps and their values.

##### Navigation

The annual diesel consumption is calculated on the basis of an average of 500 h of navigation per year, according to [36]. In the literature, the lifetime of a motor yacht is estimated between 20 and 30 years [37,38], so in the present work an average value of 25 years was set for the ship navigation phase, in accordance with other papers [36,39].

Table 4 reports the mean fuel consumption during the navigation phase for both paintings, based on experimental towing campaigns, carried out with ~2.5 m scale model boats DTMB (David Tailor Model Basin USA, Series 62-4667-1), into the Iseo Lake (Lombardy, Italy), sailing at a speed of 7.65 m/s, i.e., ~15 knots.

The towing campaigns, by data detection via IMU electronic control unit (such as speed, trim angles, pitch, roll, and yaw), strain gauge load cell (sensitivity of at least one gram of load), plus analogical detection on the towing dynamometer, were applied on clean and heavily fouled hulls, analyzing the trends of the resistance-velocity (R-V) curves for the two coatings. No differences were detected for clean boats, while experimental tests showed a better performance for tests conducted on heavily-fouled hulls painted with the EXP coating. Scaling up the performance from the model to a 120 tons motor yacht, with a hull surface of ~157.33 m^2^, equipped with a ~2.65 MW 4xVolvo D13-900 engine, the presence of a strong layer of vegetation, more detrimental for the TRD coating, results in an estimated hourly diesel consumption of approximately 41 kg/h higher than the EXP, due to a higher absorption of required power of ~200 kW. It follows that the application of EXP coating allows a significant slowdown in the vegetation growth over time, and a consequent improvement of the hydrodynamic performance of the hull, resulting in a reduction of the hourly fuel consumption of ~0.26 kg/(h × m^2^), as reported in Table 4.

Finally, data obtained on hourly specific fuel consumption, i.e., for 1 m^2^ of hull surface, were used to model the sailing phase of the motor yacht under investigation, i.e., with a hull surface of 105 m^2^.

###### Disposal

For the disposal of each material used in the production and application phase of the paints, the conditions selected on the SimaPro^®^ SW are based on the following assumptions:

- For abrasive discs, an inert landfill disposal process is considered, since they are nonhazardous wastes;
- For both plastic sheets and paper scotch, an end-of-life recycling process is considered;
- For paints, primer and solvents residues, waste from the coating stage, disposal within a hazardous waste incinerator is considered;
- For the water, necessary for the boat cleaning phase, a purification process of the sewage is considered.

Table 5 reports the items consumed during the seven steps and their values.

### 2.2. Life Cycle Costing

To carry out the economic assessment the following assumptions were made:

- all the costs must be considered before tax;
- all costs are average values referring to the year 2022;
- all the processes involved are located in Italy.

Table 6 reports the items considered for the estimation of the production costs of the two paints, based on the formulations reported in Table 1 and Table 2, considering the production of paint batches of 150 kg. While Table 7 lists the hypotheses related to the painting process (i.e., manpower, electricity, diesel, water, etc.). It is worth noting that the values reported in the latter tables are chosen according to the authors’ experience and the available information of datasheets and producers’ reports.

In order to estimate the total costs, it should be considered the consumption of the machinery, i.e., the pneumatic orbital sander with compressed air used in steps 2 and 3, and the spray gun for steps 5 and 6. Based on a mean efficiency value of 0.7 of the air compressor, as well as on an operating pressure of 6.2 bar, for both the pneumatic orbit sander and the spray painting equipment, it was possible to derive the electrical power absorbed by the machinery, based on the volumetric flow rate, as well as the absorbed energy and the overall cost. Table 8 reports the values adopted. The cost for using these two machines is the same for the two types of paint applied. Finally, the waste created during the painting process is disposed of. In Table 9, the costs for the disposal of each individual material have been calculated, divided by category.

Finally, to model the life cycle costs for 25 years of service, the presence of a sailor and a captain on the yacht for the entire annual navigation period, i.e., 500 h/y, was considered. An hourly cost of ~19.23 EUR/h for a captain, and ~9.62 EUR/h for a deckhand, respectively, was considered, in accordance with the Italian national contracts for 2022.

## 3. Results and Discussion

Environmental and economic assessments were performed according to the models of life cycle presented in the previous Section 2.1 and Section 2.2. The main results are reported and discussed in the following.

### 3.1. Environmental Analysis

The aim of this section is to evaluate the potential environmental benefit that the innovative coating can guarantee compared to traditional one. According to CML 2001 baseline method, different environmental categories were analyzed: Abiotic depletion (ADP), Acidification (AP), Eutrophication (EP), Fresh water aquatic ecotoxicity (FAETP), Global warming (GWP 100), Human toxicity (HTTP), Marine aquatic ecotoxicity (MAETP), Ozone layer depletion (ODP), Photochemical oxidation (POCP), and Terrestrial ecotoxicity (TETP).

#### 3.1.1. Production Process

**Figure 1:**
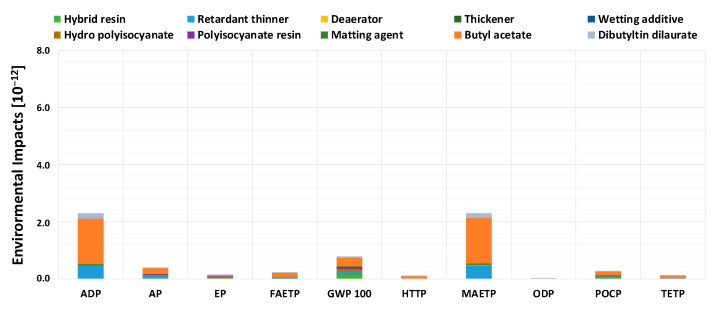
Normalized environmental impact indicators, for the production stage of 1 kg of EXP paint, divided by each of the components present.

**Figure 2:**
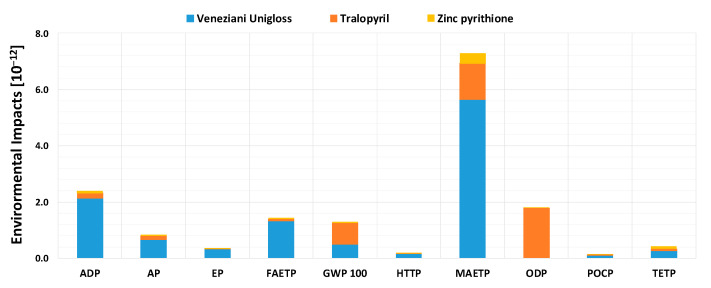
Normalized environmental impact indicators, for the production stage of 1 kg of TRD paint, divided by each of the components present.

Figure 1 and Figure 2 report the calculation of environmental impacts associated with the production of 1 kg of paint, for EXP and TRD formulations, respectively, divided by each of the components present.

It is worth noting that the impacts associated with the innovative paint in Figure 1 are much lower than traditional paint, especially for the MAETP, FAETP, and ODP damage classes, thanks also to the noncontribution of biocide additives, which are particularly hazardous to the environment [29,40]. In particular, for the ODP class, there is a highly significant decrease in the damage indicator. For the EXP paint, there is a greater contribution associated with the ADP and MAETP damage classes, mainly due to butyl acetate consumption, which is present at almost 50 wt%. The TRD formulation, on the other hand, is associated with a very high environmental impact, both for the ODP class, due to the contribution of biocides, and for the MAETP and ADP ones, due to a high contribution attributed to alkyd paint, typically used in the protection of metallic substrates [41], present ~91 wt% in the applied coating. The EXP paint thus seems to be much more eco-friendly, particularly due to substantial abatement of the major damage classes of TRD, MAETP in particular. Considering, however, that the yield of the two paints is quite different, i.e., 22.6 m^2^ for TRD versus 10.05 m^2^ for EXP, and that the latter needs to be applied twice per year, comparing the paints on the basis of the production of one kg of antifouling paint, rather than on actual annual consumption, could lead to superficial evaluations. For this reason, the overall life cycle analysis, reported in Section 3.1.3, was implemented choosing the paint coverage of 1 m^2^ of hull as functional unit.

#### 3.1.2. Navigation

Table 10 reports the calculation of the yacht’s annual fuel consumption and CO_2_ emissions during navigation, after the application of TRD and EXP coatings. These values were obtained based on the assumption of 500 h of navigation per year. It was also assumed that each liter of diesel fuel burned by the boat produces an amount of CO_2_ equal to 2.64 kg per liter of fuel, as well as a fuel density of 835 kg/m^3^.

The results reported in Table 10 shows how the adoption of the EXP coating can ensure a significant reduction in fuel consumption, ~13.7 × 10^3^ kg/y, determining an important environmental benefit, linked to a reduction of ~43.3 ton/y of CO_2_ per yacht.

#### 3.1.3. Life Cycle Analysis

**Figure 3:**
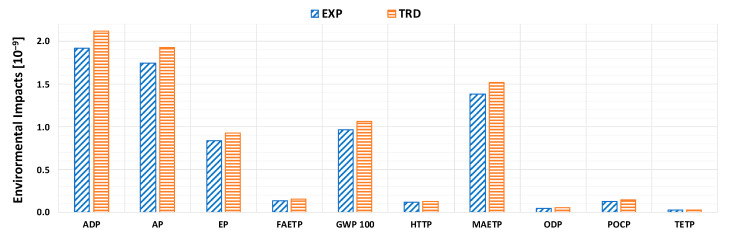
Normalized environmental impact indicators, per 1 m^2^ of hull surface per year. Please refer to Table A1 in Appendix A for more details.

In order to verify the actual greater eco-sustainability of the innovative EXP paint, compared to TRD one, the environmental impact analysis was extended to the entire life cycle, i.e., to all its phases, or production, painting, navigation and disposal, based on the quantities of paint required for the coverage of 1 m^2^ of hull per year. The overall results for the life cycle environmental impact, on annual basis, are shown in Figure 3 (please refer to Table A1 in Appendix A for more details).

The results show that switching from a traditional to an innovative paint allows a reduction by almost 10% for the most impactful damage classes, which are ADP, AP, EP, GWP 100, and MAETP for both paintings. A substantial decrease in environmental impact indicators is also observed for all other classes, except for the TETP one, for which there are no substantial differences, being EXP and TRD values comparable to each other, as well as significantly lower than for the other impacts.

**Figure 4:**
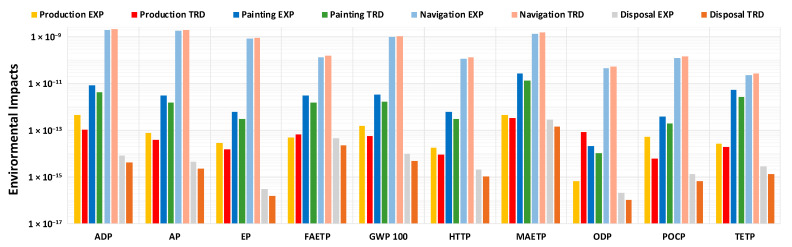
Normalized environmental impact indicators per 1 m^2^ of hull surface per year, disaggregated for each LCA stage: paint production, paint application, navigation, and disposal. Please refer to Table A2 in Appendix A for more details. Please note that both disposal stages for ADP values should be considered negative (impact reduction), while they should be considered positive for all other damage classes (impact growth). Due to the logarithmic scale, the values are reported as absolute.

In order to better understand the contribution of individual items on the overall impacts, the results of Figure 3, related to the entire life cycle, have been disaggregated into four different stages in Figure 4, i.e., production, painting, navigation and disposal (please refer to Table A2 in Appendix A for more details).

The results presented in Figure 4 show how the navigation phase is predominant over the other life cycle phases, in agreement with [36], affecting at least 99% for each of the damage classes for TRD coating, and at least 98% in the EXP coating life cycle. It follows that the ~9.6% reduction in annual consumption for EXP coating not only affects an identical reduction in impacts in the navigation phase, but how the same can occur in the overall impacts associated with the entire life cycle [36], presented in Figure 3. The only class for which the incidence of the navigation phase is lower is TETP, due to a greater comparability of impacts in the coating application phase, compared to the sailing one. In fact, the painting phase affects ~9% in the life cycle of TRD coating, while ~18% in EXP coating, due to a double application phase during the year. The reason behind this growth in relative impact can be attributed to the high value of the TETP indicator, associated with the significant consumption of compressed air, in steps 2-3-5-6 of the painting process. On the other hand, the production stage seems to affect very little in the overall impacts associated with the life cycle, with a maximum percentage impact of ~0.16% for the ODP class in case of TRD coating, due to the presence of biocides in the formulation; at the same time, a maximum value of ~0.09% is observed for the TETP class in case of EXP coating, mainly attributable to the consumption of butyl acetate. The contribution of the disposal steps seems to be even lower, compared to the overall life cycle environmental impact, with a maximum contribution for the FAETP class of ~0.01% in case of TRD coating, due to the presence of biocides in the formulation, and ~0.03% for EXP coating, mainly attributable in both cases to disposal paint remains by incineration process.

Comparing the processes with each other, based on the results in Figure 4, it can be observed that there is a general comparability between the environmental damage indicators for the production, painting, and disposal phases of the two coatings, with slightly higher values for EXP coating, for all damage classes, attributable to a double annual application process. The only exception is the ODP class, for which in the production phase TRD coating takes on values ~100 times higher. In any case, these increases in impact are quite negligible compared to the navigation phase, with a particularly significant result of almost 10% reduction in overall impact, for all main damage classes. So, through the adoption of the EXP coating, it is possible to generate a benefit effect on the environmental impact, of a considerable entity and much greater than the damage impacts of other life cycle stages; in addition, there is the possibility of reducing CO_2_ production per m^2^ of yacht hull by ~412 kg/y.

### 3.2. Economic Analysis

The aim of this section is to analyze the potential financial benefit that the proposed solution can allow in comparison with the traditional paint as antifouling coating for naval applications. The data obtained for the production of 1 kg of paint are shown in Table 11. In the latter, the comparison is intended for covering the entire surface of the hull that will be in contact with the marine environment, which in this case is about 105 m^2^. It is worth noting that by applying the coating it is estimated that 1 kg of traditional paint covers around 22.6 m^2^ of hull, while the innovative one around 10.05 m^2^. In other words, to cover the hull it is necessary to use 10.45 kg of the innovative paint against 4.65 kg of the traditional one.

As shown in Table 11, the traditional paint has a higher cost per kg than the traditional one, around 4.7 times. This is due to the high cost of the two biocides, i.e., tralopyril and zinc pyrithione, necessary to be added to the Unigloss paint. These two types of biocides give the paint the ability to prevent or eliminate the development of marine organisms on the hull of boats, through a mechanical action. However, they are released over time into the marine environment causing serious damage, as detailed in Section 3.1. Despite this, it is worth to note that the innovative paint should be applied twice per year, due to the degradation, which is two times faster than the traditional one. This means a final benefit of around 21 EUR if applying the proposed coating.

Table 12 shows the costs of the painting process, divided into the seven steps described in Section 2.1.2. The evaluation has been carried out based on the application of the coating over the full surface of the hull. It is worth to mention that for the boat transport (i.e., steps 1 and 7) and for the covering of the remaining surface not to be coated (i.e., step 4) two operators were considered. While the other activities can be carried out by a single operator.

As can be seen in Table 12, the costs for a single painting process differ only for the item related to the production of the paints themselves, with a resulting saving of 244 EUR by painting the yacht hull with the innovative solution. However, it is worth noting that the proposed alternative requires to be applied twice per year. This results in a total cost which is almost double if compared to the traditional paint.

The costs of the waste disposal are listed in Table 13. It is worth highlighting that the estimated values are the same for both paints.

As shown in Table 5, the water used during the first step by means of the pressure washer cannot be recycled since it is contaminated by the paint scraps and incrustation, but no disposal cost has been added in Table 13, as the failure to recycle water has already been factored into the painting costs in Table 12. Among the cost items, it is evident that the most influential is the abrasive waste materials disposal, with a percentage of around 91.2% of the total.

Table 14 describes the annual navigation costs due to the diesel consumption, taking into consideration an average of 500 h of navigation per year, and to the cost of labor for a sailor and a captain on board. It is important to note that the cost for the fuel consumption was calculated as average on the diesel price for the year 2022.

As reported in Table 14, the application of the innovative coating allows a saving of more than 31,000 EUR/y thanks to a reduction in fuel consumption as a consequence of the reduced hydrodynamic resistance, since the paint ensures the reduction of incrustations due to biofouling accumulation, which are considered the main factors slowing down the boats in general [42].

Finally, considering all the phases involved, from the production of the paints, to their application, disposal, and navigation, the total costs of the two antifouling coatings are shown in Table 15. For the latter, it is important to mention that the application of the innovative paint twice a year is considered in the cost calculation, while only once a year for the traditional paint.

As detailed in Table 15, each year, the application of the innovative biocide-free antifouling coating allows a saving of around 27,722 EUR if compared to the traditional one, with a reduction of 7.76% of the total costs, which increases up to almost 700,000 EUR during the service life of 25 years. The main contribution is given by the fuel saving, thanks to the improved hydrodynamics of the yacht, which is a direct consequence of the reduced accumulation of biofouling on the hull of the boat. This contribution accounts for savings of almost 780,000 EUR, over the 25-year service life of the vessel, covering the higher costs of coating application, deposited twice a year for EXP paint. However, the higher disposal and lower production costs for EXP are marginal to the overall calculation and tend to balance each other out.

## 4. Conclusions

The present work compares an innovative biocide-free antifouling coating for naval applications to a commercial paint providing a detailed and comprehensive insight of the economic and environmental benefits that arise thanks to the reduction of incrustations and toxicity to the marine environment. The major conclusions can be drawn as follows:

- the innovative paint requires two applications per year, with a total cost for production and application which is almost double to the traditional one, i.e., 7354.14 EUR against 3922.88 EUR, respectively;
- however, the proposed coating allows a significant reduction in fuel consumption, around 13.7 × 10^3^ kg/y, which consequently entails a reduction of approximately 43.3 ton/y of CO_2_ emissions per yacht and a saving of more than 31,000 EUR/y for the purchase of the fuel itself;
- the application of the innovative coating allows a reduction of the overall environmental impacts associated with the life cycle of the paint, by almost 10% for the most impactful damage classes, ensuring a greater environmental sustainability;
- the slight increase in environmental impacts in the production, painting, and disposal processes, due to a double annual application of EXP paint, is in fact negligible compared to the ~9.6% reduction in fuel consumption during the navigation phase;
- if considering a life service of 25 years, the application of the alternative paint guarantees a total saving of more than 693,000 EUR per yacht, with a reduction of 7.76% of the total costs.

In summary, the innovative biocide-free EXP formulation proves to be a valuable alternative to traditional antifouling coating for naval applications, showing an improved eco-sustainability, also avoiding the release of toxic substances into the marine environment, as well as providing significant economic savings over the entire service life of the vessel, due to increased efficiency of the antifouling action.

## Figures and Tables

**Table 1:** Formulation to produce 1 kg of the innovative biocide-free antifouling paint (EXP).

Formulation	Representative Dataset	Quantity [kg]
Silicone-polyester hybrid resin	Silikoftal^®^^1^	1.63 × 10^−1^
Retardant thinner for polyurethane paints	Butyl acetate	1.52 × 10^−1^
Deaerator for industrial coatings	Silicone product	1.36 × 10^−3^
Polyurethane-based thickener	Sikaflex^®^ PRO3 ^1^	6.80 × 10^−4^
Siloxane-based wetting additive	Tego^®^ Airex ^1^	1.36 × 10^−3^
Hydrophilic aliphatic polyisocyanate	Aliphatic isocyanates	6.27 × 10^−2^
Aliphatic polyisocyanate resin	Aliphatic isocyanates	4.63 × 10^−2^
Silica-based matting agent	Silicone product	1.99 × 10^−2^
Butyl acetate	Butyl acetate	4.97 × 10^−1^
Dibutyltin dilaurate	Butyl acetate	5.52 × 10^−2^

^1^ Items entered by authors, based on environmental impact reports provided by manufacturers.

**Table 2:** Formulation to produce 1 kg of the traditional antifouling paint (TRD), based on the addition of two biocides to the commercial paint “Unigloss”.

Formulation	Representative Dataset	Quantity [kg]
Veneziani Unigloss paint	Alkyd paint	9.09 × 10^−1^
Tralopyril	Chlorodifluoromethane	5.45 × 10^−2^
Zinc pyrithione	Zinc sulphide	3.64 × 10^−2^

**Table 3:** Analysis of the step times and consumed items for the production and painting processes. The consumption values must be intended for 1 m^2^ of hull surface, while times refer to entire stage duration. Please consider that all the steps are the same for both paints.

Step		Time [h]	Item	Consumption
1	Boat transport for painting	0.5	Diesel [L]	3.81 × 10^−2^
	Boat cleaning	2.0	LV electricity [kWh]	1.62 × 10^−1^
			Water [L]	1.14 × 10^1^
2	Paint removal	80.0	Abrasive disc [pcs] ^1^	2.86
			Compressed air [L] ^2^	2.22 × 10^4^
3	Preparation of the surface to be painted	12.0	Abrasive disc [pcs] ^3^	2.86 × 10^−1^
			Compressed air [L] ^2^	3.33 × 10^3^
4	Covering of the remaining surface	0.67	Paper scotch [kg] ^4^	3.81 × 10^−3^
			Plastic sheet [kg] ^5^	7.87 × 10^−3^
5	Primer and thinner application	3.0	Compressed air [L] ^2^	1.13 × 10^4^
			Gelshield 200 [kg]	2.20 × 10^−1^
			Thinner [kg]	3.14 × 10^−2^
6	Coating	3.0	Compressed air [L] ^2^	1.13 × 10^4^
			EXP paint [kg] ^6^	9.95 × 10^−2^
			TRD paint [kg] ^7^	4.42 × 10^−2^
7	Boat transport for sealing	0.5	Diesel [L]	3.81 × 10^−2^

^1^ Abrasive grit 60. 225 mm diameter. Corresponding to a weight of ~0.101 kg/m^2^ hull surface. ^2^ Supplied in low pressure, both for the pneumatic orbital sander and for the painting system with spray gun. ^3^ Abrasive grit 120. 225 mm diameter. Corresponding to a weight of ~0.007 kg/m^2^ hull surface. ^4^ Weight corresponding to a consumption of ~0.95 m of paper scotch, 50 mm-wide, for every m^2^ of hull. ^5^ Weight corresponding to a consumption of ~1.43 m^2^ of plastic sheet for every m^2^ of hull. ^6^ 1 kg of EXP paint is necessary to cover a hull surface of 10.05 m^2^, or 10.45 kg for the entire yacht. ^7^ According to technical datasheet, 1 kg of TRD paint is necessary to cover a hull surface of 22.60 m^2^, or 4.65 kg for the entire yacht, with a thickness of the painting of 20 µm.

**Table 4:** Analysis of the mean diesel consumption during the navigation phase, based on experimental tests conducted on heavily fouls hulls. The values must be intended for 1 m^2^ of hull surface.

Painting	Fuel Consumption	
	[kg/h]	[kg/y]
EXP	2.46	1228.30
TRD	2.72	1358.74

**Table 5:** Analysis of disposal phase. The values must be intended for 1 m^2^ of hull surface. Please consider that all the waste flows are valid for both paints.

Waste	Disposal Scenario	Quantity [kg]
Water	Purification process	1.14 × 10^1^
Plastic sheet	Recycling	7.87 × 10^−3^
Paper scotch	Recycling	3.81 × 10^−3^
Abrasive disc ^1^	Inert material landfill	1.08 × 10^−1^
Gelshield 200	Hazardous waste incineration	5.24 × 10^−3^
Thinner	Hazardous waste incineration	1.36 × 10^−3^
Paint ^2^	Hazardous waste incineration	2.10 × 10^−3^

^1^ Considering both 60 and 120 abrasive grit. ^2^ Item valid for both TRD and EXP paintings.

**Table 6:** Cost items to produce 1 kg of antifouling coating.

Coating	Item	Cost [EUR/kg]
EXP	Silicone-polyester hybrid resin	12.00
	Retardant thinner for polyurethane paints	6.18
	Deaerator for industrial coatings	19.44
	Polyurethane-based thickener	6.84
	Siloxane-based wetting additive	24.24
	Hydrophilic aliphatic polyisocyanate	12.23
	Aliphatic polyisocyanate resin	13.50
	Silica-based matting agent	12.36
	Butyl acetate	0.95
	Dibutyltin dilaurate	295.20
TRD	Veneziani Unigloss paint	20.66
	Tralopyril	900.00 ^1^
	Zinc pyrithione	900.00 ^1^

^1^ Cost estimate made by the authors, based on the purchase of the quantities of biocides needed for the production of paint batches of 150 kg, i.e., on a purchase order of ~5 to 10 kg for each product.

**Table 7:** Cost items related to the painting process.

Item	Cost
Manpower [EUR/h]	16.68
Electricity [EUR/kWh]	0.34
Diesel [EUR/L]	1.90
Water [cEUR/L]	0.14
Abrasive disc, grit 60 [EUR/disc]	0.63
Abrasive disc, grit 120 [EUR/disc]	0.69
Paper scotch [EUR/m]	0.12
Plastic sheet [EUR/m^2^]	0.61
Gelshield 200 [EUR/L]	44.00
Thinner [EUR/L]	18.00

**Table 8:** Energy consumption related to compressed air during the painting process.

Machine	Volumetric Flow Rate [L/min]	Power Consumption [kW]	Operating Time [h]	Energy [kWh]
Pneumatic sander	485	7.16	92 ^1^	658.68
Spray gun	6600	97.43	6 ^2^	584.57

^1^ Considering 80 h and 12 h, respectively, for steps 2 and 3. ^2^ Considering 3 h for each of steps 5 and 6.

**Table 9:** Disposal costs.

Waste	Type	Cost [EUR/kg]
Water	Water	0.00
Plastic sheet	Plastic packaging	0.55
Paper scotch	Paper and cardboard packaging	0.08
Abrasive disc	Abrasive waste materials	2.06
Gelshield 200	Paint scrap	1.89
Thinner	Solvent	2.24
Paint	Paint scrap	1.89

**Table 10:** Fuel consumption and related CO_2_ emissions analysis per yacht.

Item	Entire Yacht		Benefit ^1^	1 m^2^ of Hull		Benefit ^1^
	EXP	TRD		EXP	TRD	
Annual fuel consumption [10^3^ kg/y]	128.97	142.67	−13.70	1.23	1.36	−0.13
Annual CO_2_ emissions [10^3^ kg/y]	407.77	451.07	−43.30	3.88	4.30	−0.41

^1^ It is evaluated as the difference between the EXP item and the TRD one. Negative values indicate a reduction in consumption and emission.

**Table 11:** Estimated costs for the production of the paints.

Paint	Cost [EUR/kg]	Cost [EUR/yacht] ^1^	Cost [EUR/yacht/y]
EPX	21.37 ^2^	223.33	446.66 ^3^
TRD	100.59 ^2^	467.33	467.33
Benefit ^4^	−79.22	−244.00	−20.67

^1^ Calculated considering the surface of the yacht hull of around 105 m^2^. ^2^ Values obtained based on the formulations in Table 1 and Table 2, and unit costs in Table 6. ^3^ The innovative paint requires two applications per year. ^4^ It is evaluated as the difference between the EXP item and the TRD one. Negative values indicate a reduction in estimated cost.

**Table 12:** Estimated costs for the application of the paints per yacht per year.

Step		Item	Cost [EUR/yacht/y]	
			EXP	TRD
1	Boat transport for painting	Diesel	8.02	8.02
		Manpower	16.68	16.68
	Boat cleaning	LV electricity + water	7.36	7.36
		Manpower	33.37	33.37
2	Paint removal	Abrasive disc	189.90	189.90
		Pneumatic sander	192.62	192.62
		Manpower	1334.78	1334.78
3	Preparation of the surface to be painted	Abrasive disc	20.80	20.80
		Pneumatic sander	28.89	28.89
		Manpower	200.22	200.22
4	Covering of the remaining surface	Paper scotch + plastic sheet	102.90	102.90
		Manpower	22.24	22.24
5	Primer and thinner application	Gelshield 200 + thinner	978.00	978.00
		Spray gun	98.30	98.30
		Manpower	50.05	50.05
6	Coating	Spray gun	98.30	98.30
		Paint	223.33	467.33
		Manpower	50.05	50.05
7	Boat transport for sealing	Diesel	8.02	8.02
		Manpower	16.68	16.68
Number of applications per year			2	1
Total			7354.14	3922.88

**Table 13:** Estimated disposal costs of the waste collected during the seven steps of the painting process.

Waste	Type	Cost [EUR/yacht/y]	
Water	Water	0.00	
Plastic sheet	Plastic packaging	0.45	
Paper scotch	Paper and cardboard packaging	0.03	
Abrasive disc	Abrasive waste materials	23.42	
Gelshield 200	Paint scrap	1.04	
Thinner	Solvent	0.32	
Paint	Paint scrap	0.42	
Number of applications per year		EXP	TRD
		2	1
	Total	51.35	25.68

**Table 14:** Estimated navigation costs.

Item	Cost [EUR/yacht/y]		
	EXP	TRD	Benefit ^1^
Fuel	293,410.94	324,569.68	−31,158.74
Manpower	28,846.00	28,846.00	0.00
Total	322,256.94	353,415.68	−31,158.74

^1^ It is evaluated as the difference between the EXP item and the TRD one. Negative values indicate a reduction in estimated cost.

**Table 15:** Total costs per yacht per year and per 25 years of service.

Item	Cost [EUR/yacht/y]			Cost [EUR/yacht/Service Life]		
	EXP	TRD	Benefit ^1^	EXP	TRD	Benefit ^1^
Production	446.66	467.33	−20.67	11,166.50	11,683.25	−516.75
Painting ^2^	6887.48	3455.55	3431.93	172,187.00	86,388.75	85,798.25
Disposal	51.35	25.68	25.67	1283.75	642.00	641.75
Navigation	322,256.94	353,415.68	−31,158.74	8056,423.46	8835,391.99	−778,968.53
Total	329,642.43	357,364.24	−27,721.81	8241,060.71	8934,105.99	−693,045.28

^1^ It is evaluated as the difference between the EXP item and the TRD one. Negative values indicate a reduction in estimated cost. ^2^ In contrast to Table 12, the production cost of EXP or TRD paints was not considered in this item, since it was already included in the “production” item, or in the upper row of the table.

## Data Availability

Not applicable.

## Appendix A

**Table A1:** Normalized environmental impact indicators per 1 m^2^ of hull surface per year.

Damage Class	Impact Score [10^−10^]		Benefit ^1^ [10^−10^]
	EXP	TRD	
Abiotic depletion (ADP)	192.36	212.25	−19.89
Acidification (AP)	174.71	193.07	−18.36
Eutrophication (EP)	83.75	92.61	−8.86
Freshwater aquatic eco-toxicity (FAETP)	13.63	14.90	−1.27
Global warning (GWP 100)	96.11	106.10	−9.99
Human toxicity (HTP)	11.60	12.79	−1.19
Marine aquatic eco-toxicity (MAETP)	138.67	151.82	−13.16
Ozone depletion (ODP)	4.62	5.12	−0.50
Photochemical ozone creation (POCP)	12.62	13.93	−1.31
Terrestrial eco-toxicity (TETP)	2.78	2.77	0.01

^1^ It is evaluated as the difference between the EXP item and the TRD one. Negative values indicate a reduction in impacts and an effective environmental benefit.

**Table A2:** Normalized environmental impact indicators per 1 m^2^ of hull surface per year, disaggregated for each LCA stage.

Damage Class	Impact Score Production [10^−15^]		Impact Score Painting [10^−15^]		Impact Score Navigation [10^−12^]		Impact Score Disposal [10^−15^]	
	EXP	TRD	EXP	TRD	EXP	TRD	EXP	TRD
ADP	455.78	106.20	8276.09	4138.05	1914.90	2118.26	−8.51	−4.25
AP	77.71	36.97	3048.48	1524.24	1743.94	1929.13	4.53	2.26
EP	27.35	14.99	596.47	298.23	836.87	925.75	0.30	0.15
FAETP	48.39	64.10	2961.48	1480.74	133.25	147.40	43.78	21.89
GWP 100	156.41	57.07	3317.07	1658.53	957.59	1059.29	9.69	4.84
HTP	18.39	8.86	624.75	312.37	115.32	127.56	1.99	1.00
MAETP	457.73	322.93	25,286.16	12,643.08	1360.64	1505.13	279.95	139.98
ODP	0.64	79.45	20.65	10.33	46.16	51.07	0.20	0.10
POCP	52.47	6.00	374.81	187.41	125.72	139.08	1.34	0.67
TETP	26.39	19.10	5025.94	2512.97	22.79	25.22	2.70	1.35
